# Dynamics of Polarized Macrophages and Activated CD8^+^ Cells in Heart Tissue of Atlantic Salmon Infected With Piscine Orthoreovirus-1

**DOI:** 10.3389/fimmu.2021.729017

**Published:** 2021-09-16

**Authors:** Muhammad Salman Malik, Ingvild Berg Nyman, Øystein Wessel, Maria K. Dahle, Espen Rimstad

**Affiliations:** ^1^Section of Virology, Faculty of Veterinary Medicine, Norwegian University of Life Sciences, Ås, Norway; ^2^Department of Fish Health, Norwegian Veterinary Institute, Ås, Norway

**Keywords:** Atlantic salmon, cell mediated immunity, heart and skeletal muscle inflammation, macrophage polarization, Piscine orthoreovirus 1

## Abstract

Piscine orthoreovirus (PRV-1) infection causes heart and skeletal muscle inflammation (HSMI) in farmed Atlantic salmon *(Salmo salar)*. The virus is also associated with focal melanized changes in white skeletal muscle where PRV-1 infection of macrophages appears to be important. In this study, we studied the macrophage polarization into M1 (pro-inflammatory) and M2 (anti-inflammatory) phenotypes during experimentally induced HSMI. The immune response in heart with HSMI lesions was characterized by CD8^+^ and MHC-I expressing cells and not by polarized macrophages. Fluorescent *in situ* hybridization (FISH) assays revealed localization of PRV-1 in a few M1 macrophages in both heart and skeletal muscle. M2 type macrophages were widely scattered in the heart and were more abundant in heart compared to the skeletal muscle. However, the M2 macrophages did not co-stain for PRV-1. There was a strong cellular immune response to the infection in the heart compared to that of the skeletal muscle, seen as increased MHC-I expression, partly in cells also containing PRV-1 RNA, and a high number of cytotoxic CD8^+^ granzyme producing cells that targeted PRV-1. In skeletal muscle, MHC-I expressing cells and CD8^+^ cells were dispersed between myocytes, but these cells did not stain for PRV-1. Gene expression analysis by RT-qPCR complied with the FISH results and confirmed a drop in level of PRV-1 following the cell mediated immune response. Overall, the results indicated that M1 macrophages do not contribute to the initial development of HSMI. However, large numbers of M2 macrophages reside in the heart and may contribute to the subsequent fast recovery following clearance of PRV-1 infection.

## Introduction

*Piscine orthoreovirus* (PRV) infects salmonid fish and is linked to several diseases in different salmonid species (1). The subtype PRV-1 is widespread in farmed Atlantic salmon (*Salmo salar*) and is found to be the etiological cause of heart and skeletal muscle inflammation (HSMI) (2). This subtype is also associated with focal melanized changes in white skeletal muscle, commonly seen after slaughter (3). PRV-1 infection of macrophages appears to be important for the development of the melanized changes. Comparative aspects of the pathogenesis and development of melanized changes and HSMI, including the role of macrophages, has not been explored.

HSMI is a common viral disease in the marine production phase of Atlantic salmon farming in Norway, and mortality has in some cases been reported to reach 20% (4, 5). The disease is also prevalent in other Atlantic salmon farming countries including Chile (6) and Scotland (7), but with few reported outbreaks in Canada (8). Histopathological findings include moderate to severe inflammation of the epi-, myo- and endocardium layers of the heart and moderate necrosis in the red muscle tissue (9). Experimentally it has been shown that the histopathological lesions of HSMI appear about 2 weeks after the peak in the viral load during an acute PRV-1 infection (2).

In contrast, the melanized focal changes in white skeletal muscle have not been associated with clinical symptoms of disease or mortality. The condition is a rather common cause of reduced quality and declassification of the fillets. In contrast to other histopathological lesions of HSMI, the focal melanized changes are only found in the skeletal muscle, and not in the heart. The histological appearance is different from HSMI and characterized as a chronic granulomatous inflammatory reaction. The melanized focal changes appear to increase in number and severity with time after transfer of the fish from fresh to sea water (10). The chronicity of the condition indicates that it is not linked to an acute PRV-1 infection, but possibly to the persistent infection phase.

PRV belongs to the *Reoviridae* family and has a ten-segmented, double stranded RNA (dsRNA) genome packed in double layered capsid. The virus initially infects and replicates in erythrocytes, which are considered to be the major target cell population (11, 12), implying that the virus can be found in any visceral organ (8, 13). In the acute phase when there is high viremia, the virus infects cardiomyocytes and several other cell types, while in the persistent phase PRV-1 establishes a productive infection in long lived renal erythroid progenitor cells and in macrophages of Atlantic salmon (13).

The pathogenesis of HSMI is characterized by PRV-1 infection of cardiomyocytes and induction of a classical antiviral immune response that recruits CD8^+^ cytotoxic T cells (14–18). Activated cytotoxic T cells use granzymes and perforins to kill pathogen infected cells through recognition of antigen presented by major histocompatibility complex I (MHC-I) (19). Histological mapping of activated cytotoxic T cells and MHC-I positive cells and their association with PRV-1 infection could enhance the understanding of virus-specific cytotoxicity in HSMI. A transcriptomic analysis of HSMI affected heart tissue has indicated that CD4^+^ T helper cells are also present (20).

In the focal melanized changes in white skeletal muscle, PRV-1 is not found in myocytes, but in macrophages and melano-macrophages (21, 22). The macrophage responses associated with PRV-1 infection seem to be central for the pathogenesis of these melanized spots (23), i.e. the PRV-1 infection is associated with the polarization of M1 and M2 macrophage phenotypes in the pathological changes. PRV-1 infected, pro-inflammatory M1 macrophages are found in the early stages of the spot formation, while PRV-1 infected melano-macrophages of the anti-inflammatory M2 phenotype, associated with tissue repair and regeneration, are found late in the spot development (23). M1 polarized macrophages have a role in inactivation of pathogens through release of nitric oxide produced by the inducible nitric oxide synthase enzyme (iNOS2), whereas arginase-2 (Arg2) is considered as a marker for M2 type macrophages in teleost fish species (24, 25).

In the present work we studied if the PRV-1 associated macrophage polarization seen in melanized changes in white skeletal muscle, also occurred in HSMI affected heart and skeletal muscle tissue. We used material from a well characterized experimental PRV-1 challenge (2), focusing on samples collected at the time of peak virus load and at the time of maximum histopathological changes. The samples were analyzed by multiplex fluorescent *in situ* hybridization (FISH) assays in combination with gene expression by RT-qPCR. In addition, the cellular immune response was mapped by targeting MHC-I, IL-17A and CD8 positive cells.

## Materials and Methods

### Selection of Samples

Samples from a previously published PRV-1 challenge experiment were used in the present study (2). In the previous study Atlantic salmon smolts had been injected with purified PRV-1 particles (2.3x10^6^ copies/fish). The PRV-1 level had been monitored by RT-qPCR in each individual fish, as described earlier (2). The fish reached peak viral load 4 weeks post challenge (wpc) and had histopathological lesions consistent with HSMI at 6 wpc. Tissue samples from heart (n = 6) and skeletal muscle (n = 6) from 4 wpc and at 6 wpc were selected for gene expression analysis. Non-infected fish at 4 wpc (n = 3) and 6 wpc (n = 3) were included as negative controls. Heart and skeletal muscle tissue samples from fish (n=1) with peak viral load from 4 wpc (cardiac score of 0.1, scale 0-3) and 6 wpc representing peak histopathological score (cardiac score of 2.6, scale 0-3) were selected for fluorescent *in situ* hybridization analysis to compare macrophage polarization both at peak of infection and peak of histopathological lesions. Tissue from a non-infected fish of the control group was used as negative control.

### RNA Isolation and RT-PCR

Total RNA had been extracted from the heart and skeletal muscle tissues (n = 6) as previously described (2). For the present study, cDNA was synthesized from 1 µg total RNA from each tissue by using Quantitect Reverse Transcription Kit (Qiagen). Manufacturer’s guidelines were followed for the elimination of genomic DNA. Wipeout buffer (7x) was added and incubated at 42°C for 30 min with RT master-mix that includes reverse transcriptase enzyme and RNase inhibitor. A 12 µl reaction volume (15 ng cDNA input) was used for quantitative PCR using Maxima SYBR Green/ROX qPCR Master Mix (2x)-K0253 (Thermo Fisher Scientific). Thermal cycling conditions were set with initial denaturation for 10 min/95°C and 40 cycles of amplification with 15 sec/95°C, 30 sec/60°C and 30 sec/72°C. The PRV-1 load had been examined in the previous study (2). Melting curve analysis were performed to determine assay specificity. No template control (NTC) was run as a negative control on each plate. Elongation factor (EF1α) was run as a reference gene (26). Cut off value was set at Ct 35 (11). Relative fold change was measured for the genes of interest against the reference gene (EF1α) and the non-infected group (control) (**Table S1**). Specific primers targeting different genes were used for their amplification (**Table 1**).

**Table 1:** List of primers used in quantitative PCR analysis.

**Genes of interest**	Primer	Concentration	Sequence (5’-3’)	Product Size (bp)	Accession No.
***iNOS2* (** 23 **)**	Fwd	400 nM	CATCGGCAGGATTCAGTGGTCCAAT	135	XM_014214975.1
	Rev		GGTAATCGCAGACCTTAGGTTTCCTC		
***Arg2* (** 23 **)**	Fwd	400 nM	CCTGAAGGACTTGGGTGTCCAGTA	109	XM_014190234.1
	Rev		CCGCTGCTTCCTTGACAAGAGGT		
***MHC Class I* (** 27 **)**	Fwd	400 nM	CTGCATTGAGTGGCTGAAGA	175	AF504022
	Rev		GGTGATCTTGTCCGTCTTTC		
***CD8α* (** 28 **)**	Fwd	400 nM	CACTGAGAGAGACGGAAGACG	174	AY693393
	Rev		TTCAAAAACCTGCCATAAAGC		
***GzmA* (** 28 **)**	Fwd	400 nM	GACATCATGCTGCTGAAGTTG	81	BT048013
	Rev		TGCCACAGGGACAGGTAACG		
***IL-17A* (** 29 **)**	Fwd	400 nM	TGGTTGTGTGCTGTGTGTCTATGC	136	XM_014211192
	Rev		TTTCCCTCTGATTCCTCTGTGGG		
**EF1α (** 26 **)**	Fwd	500 nM	TGCCCCTCCAGGATGTCTAC	57	BG933897
	Rev		CACGGCCCACAGGTACTG		

### Statistical Analysis

Relative fold change (2- ΔΔCt formula) in gene expression level was compared between control and infected groups of fish. Nonparametric Mann-Whitney test was applied due to a low sample size in each group. GraphPad Prism version 9.0 (GraphPad Software Inc., La Jolla, CA, USA) was used for data analysis and graph layouts. A p-value *≤ 0.05* was considered as significantly different from the control.

### Histopathological Examination

Histopathological examination of heart and skeletal muscle tissues was performed to ensure that the selection criteria mentioned above were followed (2). Imaging was performed using a bright field microscope system (Carl Zeiss Light Microscopy System with Axio Imager 2 - Carl Zeiss AG, Oberkochen, Germany).

### Multiplex-Fluorescent *In Situ* Hybridization

Formalin fixed, paraffin embedded (FFPE) tissue sections (5 µm thickness) from heart and skeletal muscle tissues selected from fish with HSMI, were mounted using Superfrost plus (Thermo Fisher Scientific) slides. Sections were baked at 60°C for 2 hrs in HybEZ™ II oven (Advanced Cell Diagnostics, catalog #321720) prior to deparaffinization with absolute ethanol (100%) and fresh xylene. Initial blocking was done with hydrogen peroxide (Advanced Cell Diagnostics) for 10 min at room temperature (RT). RNAscope antigen retrieval reagent (Advanced Cell Diagnostics, catalog #322000) was used for 15 min at 99°C and slides were further incubated with RNAscope protease plus reagent for 15 min at 40°C in the HybEZ™ II oven following manufacturer guidelines. Immedge hydrophobic barrier pen (Vector Laboratories, Burlingame, CA) was used to make hydrophobic barrier around tissue areas over slides for further probe hybridization procedures.

### Multiplex *In Situ* Probes Hybridization

Simultaneous detection multiple RNA targets were performed using RNAscope^®^ Multiplex fluorescent V2 assay kit (Advanced Cell Diagnostics catalog #323100). Individual specific RNAscope probes (Listed in **Table 2**) were designed against PRV-1 L3 segment (Advanced Cell Diagnostics catalog #537451); iNOS2 (Advanced Cell Diagnostics catalog #548391); Arg2 (Advanced Cell Diagnostics catalog #548381) CD8α (Advanced Cell Diagnostics catalog #836821); Granzyme A (Advanced Cell Diagnostics catalog #836841); MHC-I (Advanced Cell Diagnostics catalog #836831) and IL-17A (Advanced Cell Diagnostics catalog #836861). Peptidylpropyl Isomerase B- (PPIB) (Advanced Cell Diagnostics, catalog #494421) was taken as a positive control for RNA integrity of the samples and dihydrodipicolinate reductase (DapB) from *Bacillus subtilis* (Advanced Cell Diagnostics catalog #310043) was taken as a negative control for cross-reactivity and background.

**Table 2:** Probes used in FISH assays.

Probe		Target Region (bp)	Fluorophores	Emission/Excitation Wavelength (nm)	Channel*
**Target**	*PRV-L3*	415–1379	Opal 520 (FP1487001KT)	494/525	C1
	*iNOS2*	2–949	Opal 620 (FP1495001KT)	588/616	C2
	*Arg2*	1332–2053	Opal 690 (FP1497001KT)	676/694	C3
	*CD8α*	8-1033	Opal 620 (FP1495001KT)	588/616	C2
	*GzmA*	3-1088	Opal 690 (FP1497001KT)	676/694	C3
	*MHC-I*	2-2321	Opal 620 (FP1495001KT)	588/616	C2
	*IL-17A*	86-486	Opal 690 (FP1497001KT)	676/694	C3
**Control**	*PPIB*	20–934	Opal 520 (FP1487001KT)	494/525	C1
	*DapB*	414–862	Opal 520 (FP1487001KT)	494/525	C1

*Channel indicates excitation and emission properties for the specific fluorophores. Accession numbers: PRV-L3- KY429945; PPIB- NM_001140870; DapB- EF191515. For the other genes the acc. nos. are listed in **Table 1**.

As per manufacturer’s instructions, all probes were mixed and hybridized to each section for 2 hrs at 40°C in the HybEZ™ II oven. Signal amplification was done by applying series of amplifier reagents (Amp1-Amp3) included in the kit. A 1:1500 diluted Opal fluorophores (Akoya Biosciences, CA, United States) in tyramide signal amplification (TSA) buffer (Advanced Cell Diagnostics catalog #322809) were assigned separately to each probe with different excitation and emission spectrum to produce a specific output signal. RNAscope^®^ multiplex Fluorescent Detection Reagents v2 (catalog #323110) were used to develop and block each probe sequentially. DAPI (fluorescent DNA stain) was used as a counter stain for 30 sec at RT. About 1-2 drops of Prolong Gold antifade mounting reagent (Thermo Fisher Scientific) were used to mount cover slides. Confocal microscopy was performed using TCS SP8 gSTED confocal microscope (Leica microsystems GmbH, Mannheim, Germany).

## Results

### Localization of M1 and M2 Polarized Macrophage Populations

Heart and skeletal muscle tissues from an experimental PRV-1 challenge were used. Samples were selected from the key time points i.e. peak virus load (4wpc), and maximum histopathological changes (6 wpc) (**Figure S1**).

**Figure 1:**
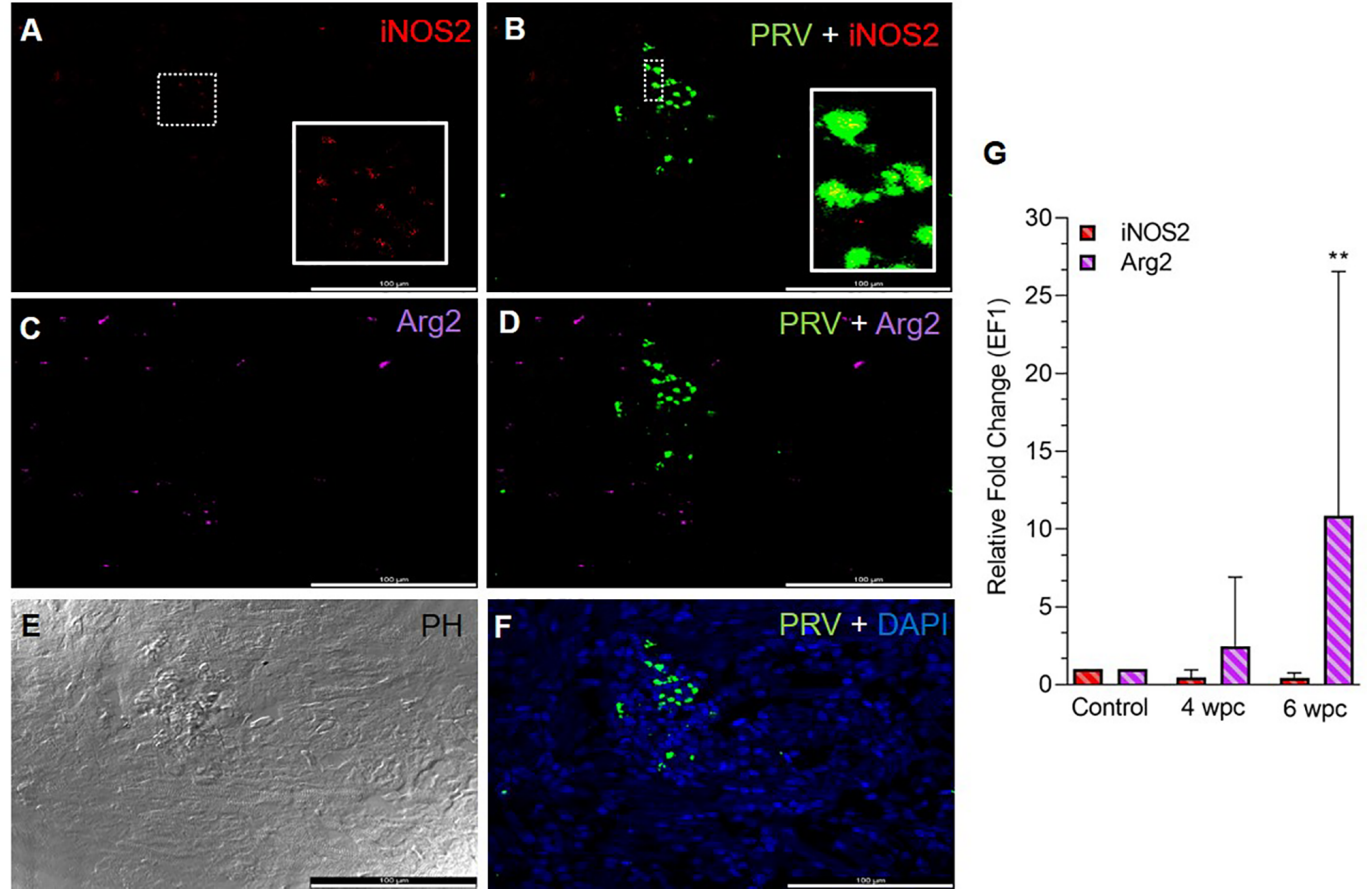
Fluorescent *in situ* hybridization (FISH) of iNOS2, Arg2 and PRV-1 **(A–F)** in heart tissue at the peak of HSMI (6 wpc). **(A)** A limited number of iNOS2 (red) positive M1 macrophages were detected. **(B)** Merged image showing modest co-localization of PRV-1 and iNOS2 (inset). **(C)** Widely distributed Arg2 (purple) positive M2 macrophages. **(D)** Merged image does not show co-localization of PRV-1 and Arg2-positive M2 macrophages. **(E)** Phase contrast image showing aggregated blood cells in the stratum spongiosum layer. **(F)** PRV-1 (green) infected cells amongst clustered blood cells. Cellular nuclei stained with DAPI (blue) (Scale Bar = 100 µm). **(G)** Relative fold change expression (medians) of iNOS2 and Arg2 at 4 wpc and 6 wpc normalized against EF1α expression. (*) shows significant difference from the control (**p < 0.01).

In the heart, a few iNOS2 positive cells (assumed M1 macrophages) were spotted in the spongiosum layer both at the peak in viremia (**Figure S2**) and at the peak in HSMI pathology (**Figure 1A**) and were localized particularly in aggregations of blood cells. iNOS2 stained cells were not found in the epicardial and compactum layers (**Figure S2**). Several of the iNOS2 positive macrophages also stained for PRV-1 (**Figure 1B**). Numerous Arg2 specific cells (assumed M2 type macrophages) were detected in heart at both time points (**Figures 1C**, **S2**), but with a more abundant appearance at 6 wpc. There was no staining of PRV-1 in Arg2 positive M2 macrophages (**Figures 1D**, **S2**). Heart tissue from both time points had a lower number of M1 polarized macrophages compared to M2 polarized macrophages, as estimated through FISH (**Figures 1**, **S2**). PRV-1 infected cells were widely scattered in the layers of epicardium, compactum and spongiosum in infected fish at both sampling points (**Figures 1F**, **S2**). The FISH results were in accordance with the transcript expression levels observed by RT-qPCR where the iNOS2 expression was low, while the Arg2 expression was upregulated (approximately 11 folds, p< 0.01) in heart tissue at 6 wpc (**Figure 1G**).

**Figure 2:**
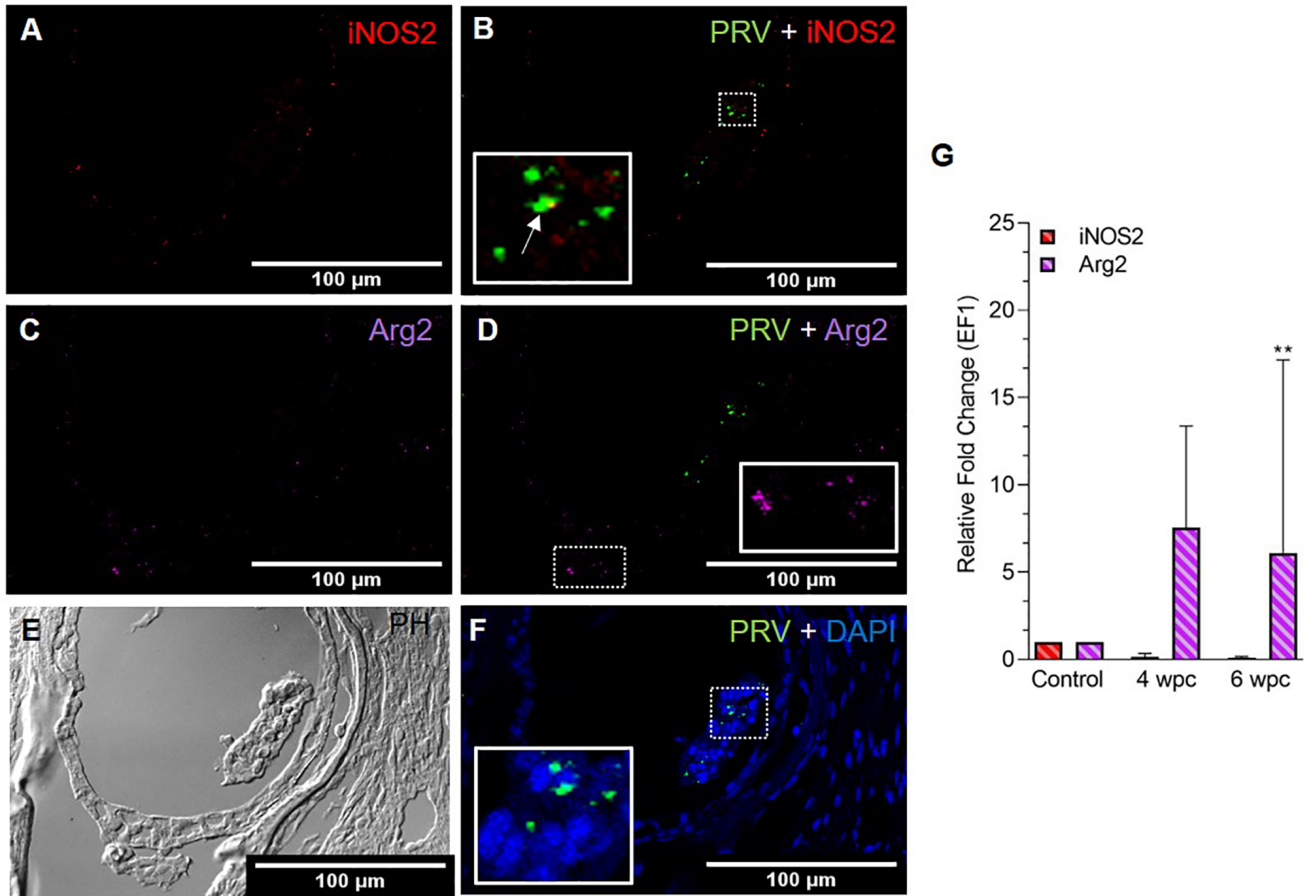
Fluorescent *in situ* hybridization (FISH) of iNOS2, Arg2 and PRV-1 **(A–F)** in skeletal muscle at the peak of HSMI (6 wpc). **(A)** Few iNOS2 (red) positive M1 macrophages were detected. **(B)** Merged image showing co-localization (yellow in inset) of PRV-1 and iNOS2-positive M1 macrophages. **(C)** Sporadic presence of Arg2 (purple) positive M2 macrophages **(D)** Merged image does not show co-localization of PRV-1 and Arg2 **(E)** Phase contrast image showing aggregated blood cells in a vessel in the center. **(F)** A limited number of PRV-1 infected cells (green) were found (Scale Bar = 100 µm). **(G)** Relative fold change expression (medians) of iNOS2 and Arg2 at 4 wpc and 6 wpc normalized against EF1α. (*) show significant difference from the control (**p < 0.01).

In skeletal muscle tissue at 4 wpc, dispersed M1 and M2 macrophage populations were observed (**Figure S3**). At 6 wpc there were few iNOS2 positive M1 macrophages detected around infected blood cells (**Figure 2A**), but the majority of the M1 macrophages were negative for PRV-1 (**Figure 2B**). A similar pattern was observed for Arg2 specific staining, with no co-localization of PRV-1 and Arg2 (**Figures 2C, D**). Only modest staining of PRV infected cells were seen in skeletal muscle (**Figure 2F**). There was a low level of iNOS2 expression, however, the Arg2 level was upregulated at 4 wpc (approximately 8 folds) but significantly upregulated at 6 wpc (6 folds, p<0.01) (**Figure 2G**).

In non-infected control fish, Arg2 positive M2 macrophages were present in heart tissue but not found in skeletal muscle (**Figures S4**, **S5**). No iNOS2 positive M1 macrophages were seen in neither heart nor skeletal muscle of these fish.

### Localization of CD8+ Cells in Heart

**Figure 3:**
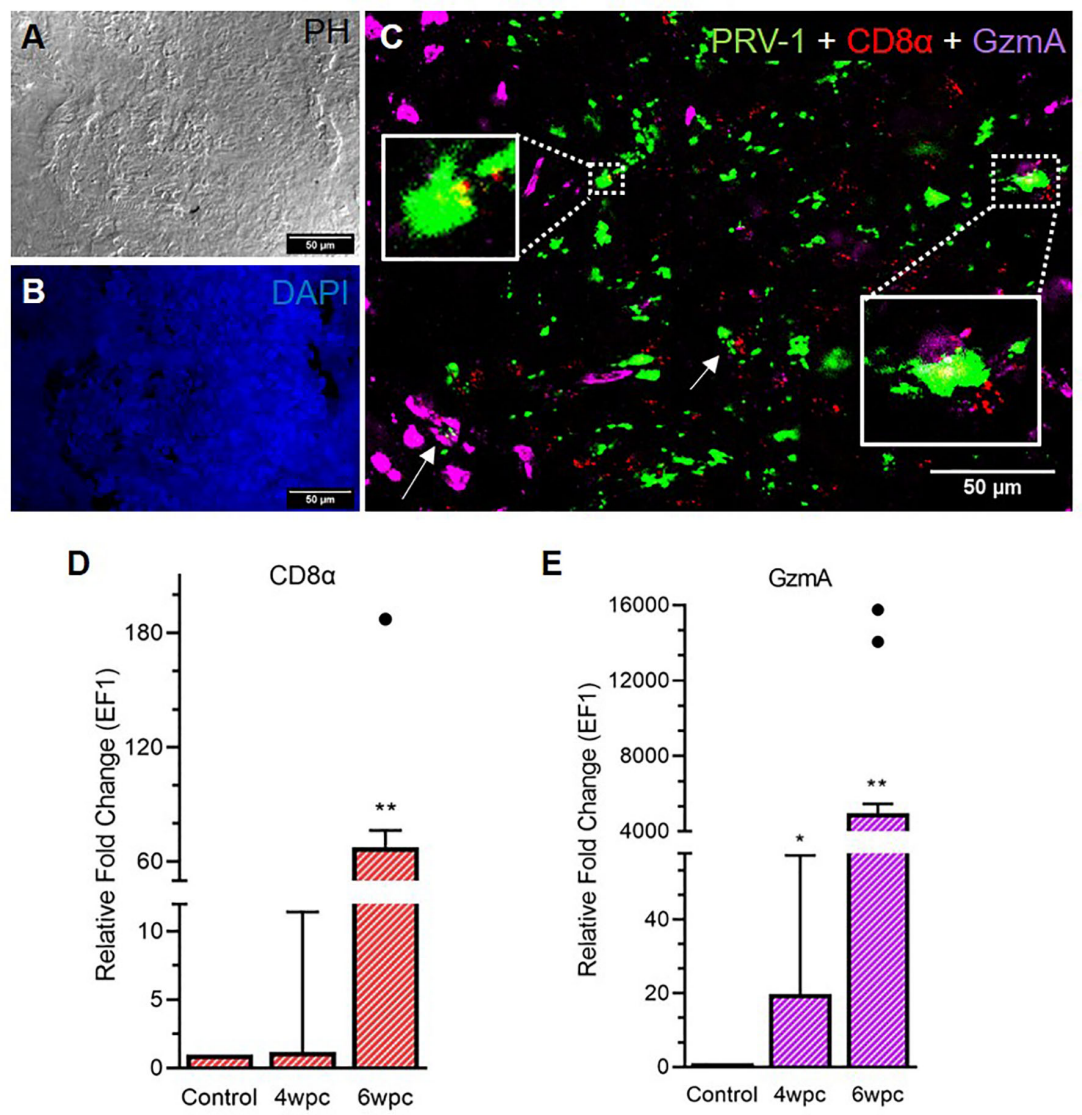
Fluorescent *in situ* hybridization (FISH) of PRV-1, CD8α and GzmA in heart tissue at peak of HSMI associated histopathological changes (6 wpc). **(A)** Phase contrast image showing blood cells in the spongiosum. **(B)** Nuclei stained with DAPI (Blue). **(C)** Merged image showing co-localization of PRV-1 (green) with CD8+ (red) cells and cells expressing GzmA (purple) (arrows, yellow in inserts). GzmA stained cells were prominent in areas with dense PRV-1 staining (Scale Bar = 50 µm). **(D, E)** Relative fold change expression (medians) of CD8α and GzmA at 4 wpc and 6 wpc normalized against EF1α. Dots show outlier values in the respective group of fish. (*) shows significant difference from the control (*p < 0.05, **p < 0.01).

A strong influx of CD8+ cells was noted at 6 wpc (**Figure 3**) whereas low to moderate level of CD8^+^ cells in the heart were detected at 4 wpc (**Figure S6**). CD8^+^ cells were abundant in the *stratum spongiosum* compared to epicardium and compactum layer. At 4 wpc, CD8^+^ cells localized around PRV-1 infected cardiomyocytes. No co-localization of PRV-1 and CD8 were seen in the same cells, but PRV-1 infected cardiomyocytes co-localized with CD8^+^ cells was a typical feature. CD8^+^ cells were found widely distributed in the different heart regions at 6 wpc but were particularly abundant in areas with dense PRV-1 staining and co-localized with PRV-1 infected cells (**Figure 3C**). CD8α gene expression showed a minor increase at 4 wpc (1.2-fold) but was significantly increased at 6 wpc (67-fold, p < 0.01) (**Figure 3D**). Upregulation of CD8α in heart at 6 wpc correlated in time with a moderate decline in PRV-1 RNA levels (**Figures 3D**, **S7**).

Granzyme A (GzmA) positive cells were prominent in heart tissue in areas with dense PRV-1 staining (**Figure 3C**). GzmA transcripts were highly increased at 6 wpc (approximately 5000 folds, p < 0.01), indicating increased cytotoxic activity (**Figure 3E**).

**Figure 4:**
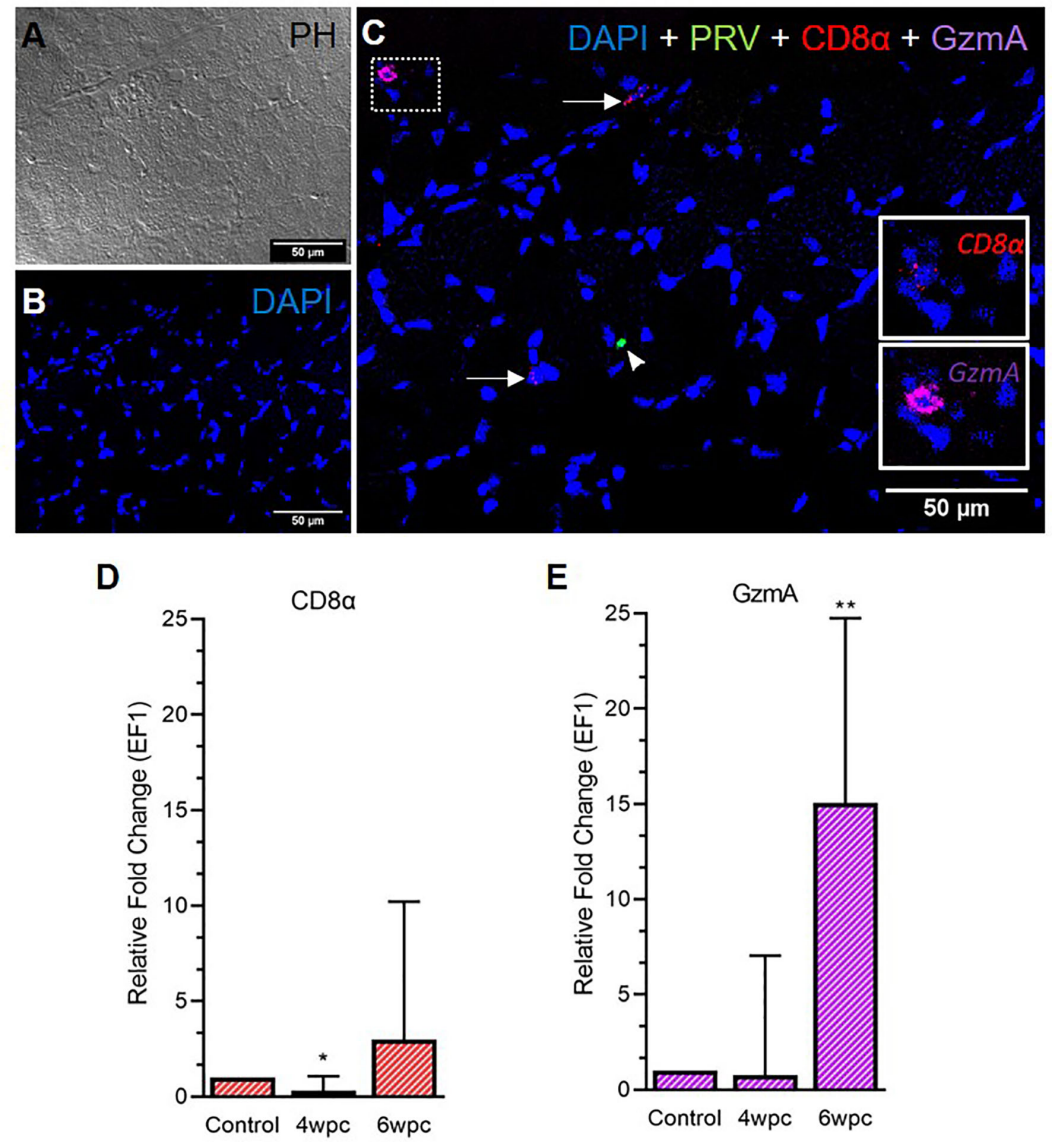
Fluorescent *in situ* hybridization (FISH) of CD8α, GzmA and PRV-1 in skeletal muscle at the peak of HSMI (6 wpc). **(A)** Phase contrast image showing structures of myocytes. **(B)** Nuclei stained with DAPI (Blue). **(C)** Merged image showing presence of PRV-1 (green) infected cell (arrowhead), and CD8+ cells (insets). Some cells were stained positive only for CD8α and GzmA specific transcripts (arrows) (Scale Bar = 50µm). **(D, E)** Relative fold expression (medians) of CD8α and GzmA at 4 wpc and 6 wpc normalized against EF1α. (*) shows significant difference from the control (*p < 0.05, **p < 0.01).

In the skeletal muscle tissue, the number of CD8^+^ cells was almost negligible at 4 wpc (**Figure S8**) and cells were sporadically present at 6 wpc (**Figure 4**). No co-localization of PRV-1 infected cells and CD8^+^ cells was detected (**Figure 4C**). Expression analysis showed a significant downregulation of CD8α expression level at 4 wpc (p < 0.05) but a slightly upregulated expression level at 6 wpc (3-fold, not statistically significant) (**Figure 4D**). GzmA positive cells were sporadically present in skeletal muscle at 6 wpc, but no co-localization with PRV-1 was seen (**Figure 4C**). Gene expression of GzmA showed significant upregulation at 6 wpc (15-fold, p < 0.01) (**Figure 4E**).

### PRV-1 in MHC-I Positive and Th17 Cells

**Figure 5:**
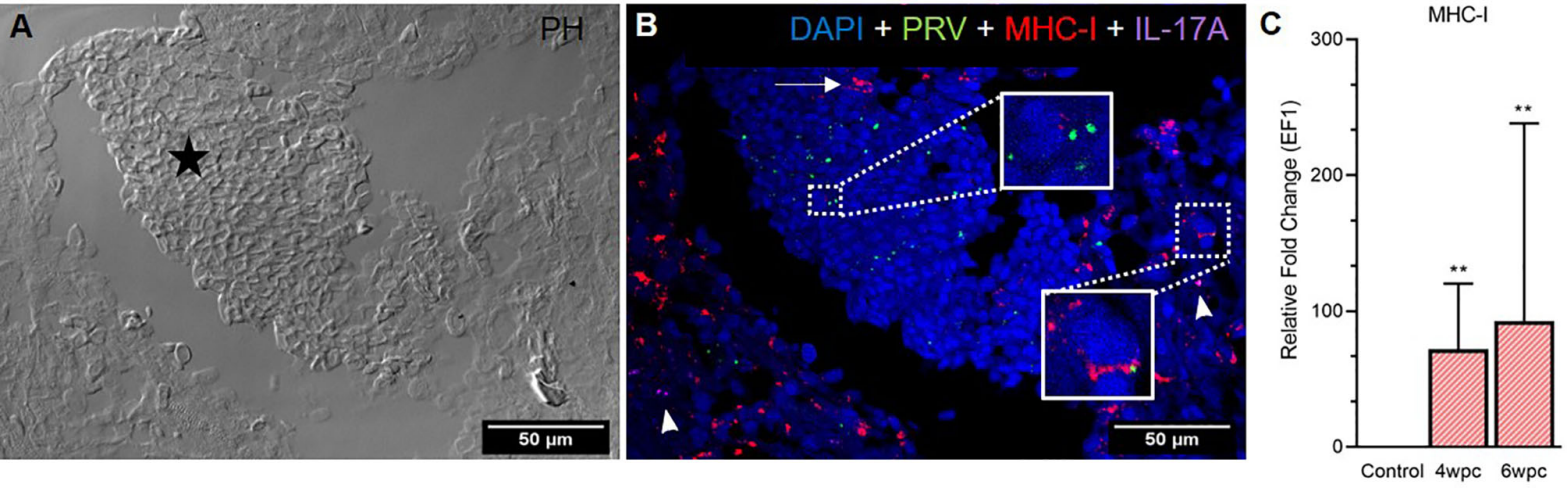
Fluorescent *in situ* hybridization (FISH) of PRV-1, MHC-I and IL-17A in heart tissue at peak of HSMI (6 wpc). **(A)** Phase contrast image showing an area with aggregated blood cells (star) in the spongiosum layer. **(B)** Merged image showing presence of PRV-1 (green) in cardiomyocytes and clotted blood cells, partially co-localizing with MHC-I (red) positive cells (insets). Some MHC-I positive cells in the aggregated blood did not co-stain for PRV-1 (arrow). IL-17A (purple) positive cells were sporadically scattered in the tissue (arrowhead). Nuclei stained with DAPI (Blue) (Scale Bar = 50 µm). **(C)** Relative fold change expression (medians) of MHC-I at 4 wpc and 6 wpc normalized against EF1α. Asterisk (*) shows significantly difference from the control (**p < 0.01).

In heart, modest co-staining of PRV-1 and MHC-I in the same cells was observed throughout the tissue at both peak of infection (**Figure S9**) and maximum HSMI pathology (**Figure 5**). Within aggregated blood cells, PRV-1 partially co-localized with MHC-I in the same cells both at 4 wpc (**Figure S9**) and 6 wpc (**Figure 5B**). However, some MHC-I expressing cells in the aggregated blood did not stain for PRV-1. Significant upregulation of MHC-I was detected at both 4 wpc (72-fold, p<0.01) and 6 wpc (93-fold, p<0.01), compared to control.

The Th17 T-helper cells of Atlantic salmon produce the proinflammatory cytokine IL-17A (29–32). In heart, specific staining showed the presence of a few IL-17A-positive cells at both samplings, however, no pattern of co-localization with PRV-1 was observed (**Figure 5B**), and gene expression analysis showed a slight downregulation (not statistically significant) of IL-17A in heart after PRV-1 infection (**Figure S10**).

**Figure 6:**
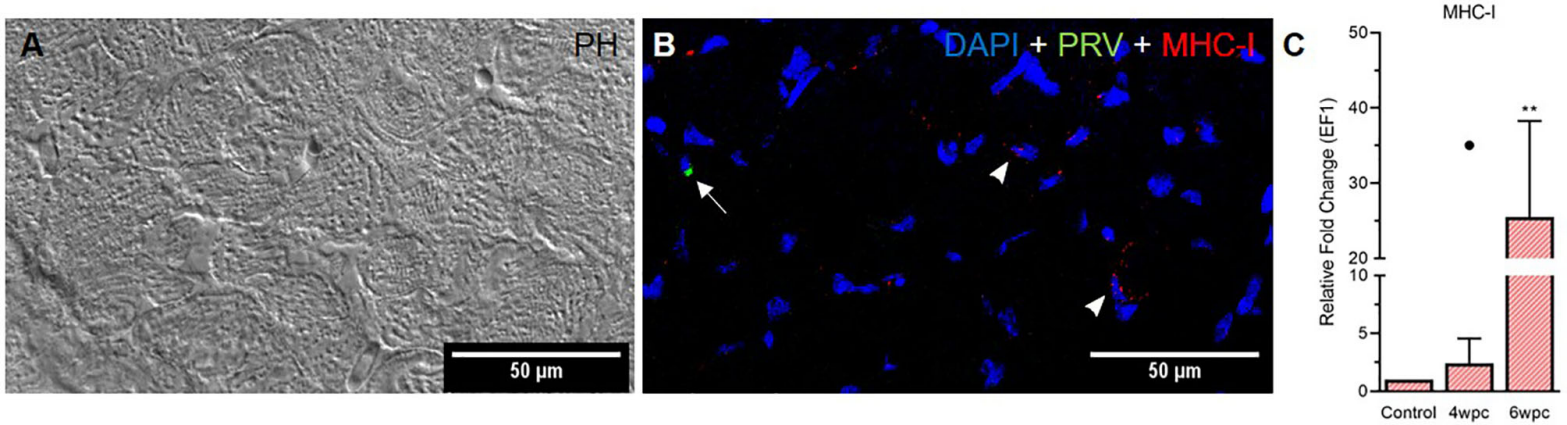
Fluorescent *in situ* hybridization (FISH) of PRV-1 and MHC-I in skeletal muscle at peak of HSMI associated histopathological changes (6 wpc). **(A)** Phase contrast image showing myocyte structure **(B)** Merged image showing MHC-I (red) positive cells (arrowheads) and PRV-1 (green) infected cells (arrow). Nuclei stained with DAPI (Blue) (Scale Bar = 50 µm). **(C)** Relative fold change expression (medians) of MHC-I at 4 wpc and 6 wpc normalized against EF1α. Dot sign shows outlier value from the respective fish group. (*) shows significantly difference from the control (**p < 0.01).

In skeletal muscle tissue MHC-I positive cells were dispersed between myocytes at 4 wpc (**Figure S11**) and 6 wpc (**Figures 6A, B**). A few PRV-1 positive cells were also present, but none of these cells were also positive for MHC-I. Expression analysis showed significant upregulation of MHC-I at 6 wpc (27-fold, p<0.01) (**Figure 6C**) which correlated with a drop in the PRV-1 level. In contrast to the heart tissue, no IL-17A specific transcripts could be detected in the skeletal muscle by FISH and RT-qPCR.

## Discussion

The aim of this study was to map the cellular immune responses in heart and skeletal muscle tissues from PRV-1 infected fish developing HSMI, by using FISH, targeting iNOS2 and Arg2 for M1 and M2 polarized macrophages respectively, with additional detection of MHC-I, IL-17A, CD8, and GzmA. The focus was set on the peak in virus load (4 wpc) and the maximal histopathological changes in the heart (6 wpc), using material from a previously performed PRV-1 experimental challenge. In accordance with the relative limited pathological changes of the skeletal muscle, the findings were in general more pronounced in heart, however the findings in the two organs were in accordance with each other.

We found that the Arg2-positive, tissue repair associated M2 type macrophages were the dominant type of macrophages in heart tissue both at 4 wpc and at 6 wpc. On the other hand, HSMI affected heart tissue showed a low presence of the classically activated M1 macrophage phenotype, based on iNOS2 staining. These results were corroborated by mRNA expression analyses by RT-qPCR. We did not observe presence of PRV-1 in M2 macrophages, and only a limited number of the M1 macrophages stained for PRV-1.

Contrasting these findings is the dominance of PRV-1 infected, pro-inflammatory M1 polarized macrophages reported from the early stages of melanized focal changes in skeletal muscle of Atlantic salmon (23). In the later stages of the melanized focal changes the anti-inflammatory M2 phenotype dominates, but still many of these M2 cells are PRV-1 infected (23). In the present study, tissue repair associated M2 macrophages were frequent in heart tissue both at 4 wpc and 6 wpc. At 4 wpc the histopathological changes had not yet developed in the heart, and FISH of hearts from naive fish showed that they also harbored M2 positive cells. That heart tissues harbor a large number of Arg2-positive M2 macrophage-like cells is in line with previous findings (13), and indicate the potential of the heart for healing *via* macrophage mediated repair mechanism (33). The presence of self-renewing tissue macrophages, seeded during embryonic hematopoiesis, makes the repair of heart tissue in fish a well-organized process (34). The macrophage M1 and M2 polarization is a dynamic process (35), and in line with this the pro-inflammatory M1 phenotype dominates early in the pathogenesis of melanized focal changes in skeletal muscle while the M2 phenotype dominates in the late stages (23). Both macrophage phenotypes can be infected by PRV-1 (23). The M1 macrophages with their high production of iNOS2 enzyme are inducers of melanin production (36), and in HSMI hearts there are only sporadic tiny patches of melanin present. The M2 macrophages found in heart did not stain for PRV-1 RNA, i.e. there was no indication of PRV-1 infection of this cell type in HSMI. Together this indicate a lack of a prominent role of M1 and M2 macrophages in development of HSMI. It can be speculated whether the differences in HSMI pathogenesis and development of melanized spots are caused by properties of the virus strain, i.e. different strains of orthoreoviruses have been shown to vary in their ability to productively infect macrophages (37). The difference in HSMI pathogenesis and development of melanized spots could also be associated to variance in resident and recruited macrophage populations in heart tissue and white skeletal muscle or related to acute *versus* persistent infection.

The co-localization of PRV-1 infected cells with CD8+ cells, and the co-staining of PRV-1 and MHC-I in the same cells enhances the understanding of the pathogenesis of HSMI. Gene expression analysis supported the *in situ* expression of CD8^+^ cell marker, and highly upregulated CD8α expression was detected in heart, in line with earlier studies (17). Specific targeting of the PRV-1 infected cells by cells producing GzmA was indicated by the co-localization with PRV-1 infected cells. GzmA specific transcripts are primarily produced by CD8^+^ T-cells, but can also be detected in natural killer cells (38). In contrast to heart, skeletal muscle tissue exhibited moderate recruitment of CD8^+^ cells and these did also not co-localize with PRV-1 infected cells.

Atlantic salmon erythrocytes show high expression of MHC-I after PRV-infection (14). MHC-I expressing cells also positive for PRV-1 were abundant and found associated with high numbers of GzmA positive cells. This finding suggests that antigen presentation to cytotoxic T cells is important for the elimination of PRV-1 from the heart. The high number of M2 macrophages indicates a potential of the heart tissue for rapid recovery. The PRV-1 level correlated inversely with high CD8^+^ cell activity and MHC-I expression in heart.

## Conclusion

The combination of FISH and RT-qPCR analysis confirmed that a strong CD8^+^ mediated immune response is important in the pathogenesis of HSMI, and demonstrated the co-localization of PRV-1 infected, MHC-I positive cells with CD8 and Granzyme A-positive cells in heart. The results did not indicate a prominent role of M1 polarized macrophages in the initial development of HSMI in Atlantic salmon however, the large population of M2 polarized macrophages resident in heart tissue indicates a prominent role of these cells in the rapid recovery after HSMI.

## Data Availability Statement

The raw data supporting the conclusions of this article will be made available by the authors, without undue reservation.

## Ethics Statement

The animal study was reviewed and approved by Animal Care and Use Committee/IACUC and NARA (permit number 7487) according to the European Union Directive 2010/63/EU and Norwegian regulation FOR-2015-06-18-761.

## Author Contributions

Conceptualization, MM and ER. Methodology, MM, IN, and ØW. Software, MM. Validation, MM, ØW, and MD. Formal analysis, MM, ØW, MD, and ER. Investigation, MM, ØW, and ER. Resources, ER. Data curation, MM and IN. Writing—original draft preparation, MM and ER. Writing—review and editing, ØW, MM, MD, and ER. Visualization, MM and IN. Supervision, ER, MD, and ØW. Project administration, ER. Funding acquisition, ER. All authors contributed to the article and approved the submitted version.

## Funding

The study was supported by the Research Council of Norway, grant 280847/E40 (ViVaAct) and by Norwegian University of Life Science.

## Conflict of Interest

The authors declare that the research was conducted in the absence of any commercial or financial relationships that could be construed as a potential conflict of interest.

## Publisher’s Note

All claims expressed in this article are solely those of the authors and do not necessarily represent those of their affiliated organizations, or those of the publisher, the editors and the reviewers. Any product that may be evaluated in this article, or claim that may be made by its manufacturer, is not guaranteed or endorsed by the publisher.

## Supplementary Material

Supplementary Figure 1 Histopathological overview of heart **(A, B)** and skeletal muscle **(C, D)** tissues at 4 and 6 wpc. **(A)** Heart section without cardiac lesions at 4 wpc. **(B)** Severe cardiac lesions at 6 wpc in all three layers of the heart (arrowheads). **(C)** Intact and conformed myocytes with minor presence of melanin (shown in inset), 4 wpc **(D)** Overall, mild degeneration with presence of small hemorrhages (shown in inset) with thin melanin deposits (arrowhead), 6 wpc. Scale bar = 50 µm.

Supplementary Figure 2 *Fluorescent in situ hybridization (FISH) of iNOS2, Arg2 and PRV-1 in HSMI (heart) at 4 wpc*. **(A)** Few iNOS2 (red) positive M1 macrophages were detected. **(B)** Merged image showing co-localization of some PRV-1 in M1 macrophages (insets). **(C)** Widely distributed Arg2 (purple) positive M2 macrophages. **(D)** Merged image showing no co-localization of PRV-1 in M2 macrophages. **(E)** Phase contrast image showing all different layers of the heart i.e. epicardium (white arrow), compactum (black arrowhead) and spongiosum (white arrowhead). **(F)** PRV-1 (green) infected cells scattered in all different layers of the heart. Nuclei stained with DAPI (blue). Scale Bar = 100 µm.

Supplementary Figure 3 *Fluorescent in situ hybridization (FISH) of iNOS2, Arg2 and PRV-1 in HSMI (skeletal muscle) at 4 wpc*. **(A)** Phase contrast image showing structure of myocytes. **(B)** Merged image showing PRV-1 infected cells (arrows) surrounded by M1 (arrowheads) and M2 macrophages (dotted circle). No co-localization of PRV-1 was observed in the M1 and M2 stained cells. Scale bar = 100µm.

Supplementary Figure 4 Skeletal muscle tissue from un-infected control fish collected at a time corresponding to 4 wpc for the PRV-1 challenged fish. **(A)** No PRV-1 (green) detection, **(B)** No iNOS2 (red) positive cells (M1) were detected, **(C)** No Arg2 (purple) positive cells (M2) were detected. **(D, E)** Nuclei were stained with DAPI (blue) and phase contrast image shows myocytes. Scale bar = 100µm.

Supplementary Figure 5 Heart tissue from un-infected control fish collected at a time corresponding to 4 wpc for the PRV-1 challenged fish. **(A)** No PRV-1 (green) detection, **(B)** No iNOS2 (red) positive cells (M1). **(C)** Phase contrast image. **(D)** Cellular nuclei were stained with DAPI (blue). **(E, F)** Few Arg2 (purple) positive cells in the heart (insets). Scale bar = 100µm.

Supplementary Figure 6 *Fluorescent in situ hybridization (FISH) of PRV-1, CD8α and GzmA in HSMI (Heart) at 4 wpc*. **(A)** Phase contrast image showing structure of epi- (e), compactum (myo- (m)) and spongiosum layer. **(B)** Merged image showing no-colocalization of PRV-1 (green) but presence of few CD8^+^ (red) cells at the infected site (inset), whereas granzyme A (purple, arrows) specific transcripts are distributed elsewhere. Scale bar = 100 µm.

Supplementary Figure 7 RT-qPCR data showing median (line) PRV-1 level (red) in **(A)** heart and **(B)** skeletal muscle tissues at peak viral load (4 wpc) and peak pathological changes (6 wpc). Uninfected fish from each timepoint was included as a control fish.

Supplementary Figure 8 *Fluorescent in situ hybridization (FISH) of PRV-1, CD8α and GzmA in HSMI (skeletal muscle) at 4 wpc*. **(A)** Phase contrast image showing myocytes structure. **(B)** Merged image showing PRV-1 (green) infected cells (inset) and sporadic presence of granzyme A (purple) specific cells (arrows). No CD8^+^ cells (red) were detected at 4 wpc. Scale bar = 100 µm.

Supplementary Figure 9 *Fluorescent in situ hybridization (FISH) of PRV-1, MHC-I and IL-17A in HSMI (Heart)* at 4 wpc. **(A)** Phase contrast image showing structure of epi- (e), compactum **(C)** and spongiosum layer. **(B)** Merged image showing partial co-localization of PRV-1 (green) in MHC-I (red) positive cells. Very few IL-17A (purple) specific transcripts were detected (inset). Scale bar = 100 µm.

Supplementary Figure 10 Relative fold change expression (medians) of IL-17A at 4 wpc and 6 wpc in the heart tissue normalized against EF1α.

Supplementary Figure 11 *Fluorescent in situ hybridization (FISH) of PRV-1, MHC-I and IL-17A in HSMI (skeletal muscle)* at 4 wpc. **(A)** Phase contrast image showing myocytes structure. **(B)** Merged image showing PRV-1 (green) infected cells (inset) surrounded by MHC-I (red) cells. Scale bar = 100 µm.

Supplementary Table 1 Ct values for samples.
