# Supplementary figures and images for: Dynamics of Polarized Macrophages and Activated CD8+ Cells in Heart Tissue of Atlantic Salmon Infected With Piscine Orthoreovirus-1
Source: Front Immunol. 2021 Sep 16;12:729017. doi: 10.3389/fimmu.2021.729017 (PMC8481380; doi:10.3389/fimmu.2021.729017)

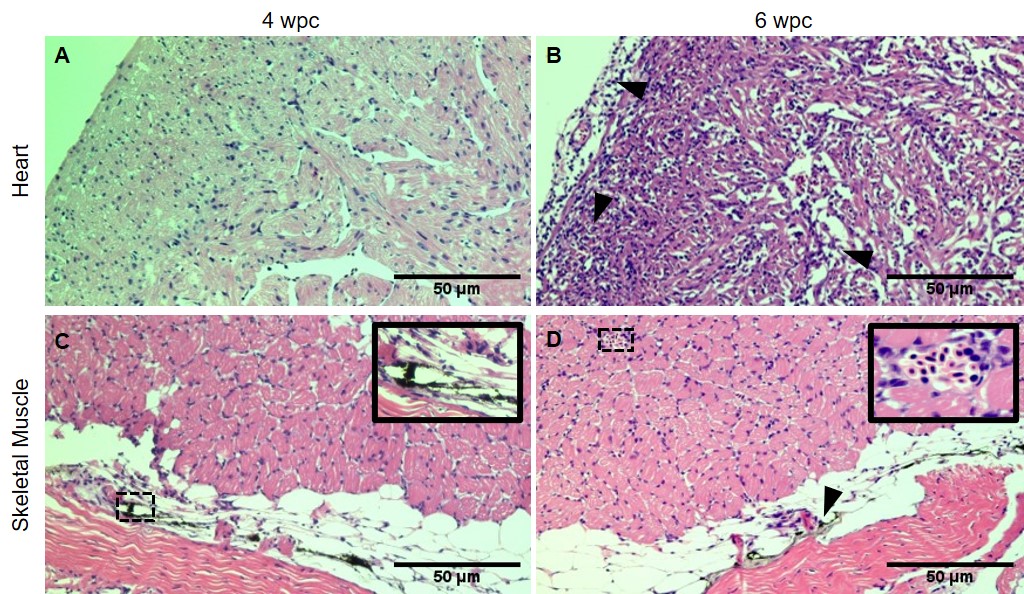

Supplement: Supplementary Figure 1 — Histopathological overview of heart (A, B) and skeletal muscle (C, D) tissues at 4 and 6 wpc. (A) Heart section without cardiac lesions at 4 wpc. (B) Severe cardiac lesions at 6 wpc in all three layers of the heart (arrowheads). (C) Intact and conformed myocytes with minor presence of melanin (shown in inset), 4 wpc (D) Overall, mild degeneration with presence of small hemorrhages (shown in inset) with thin melanin deposits (arrowhead), 6 wpc. Scale bar = 50 µm. [file Image_1.jpeg]

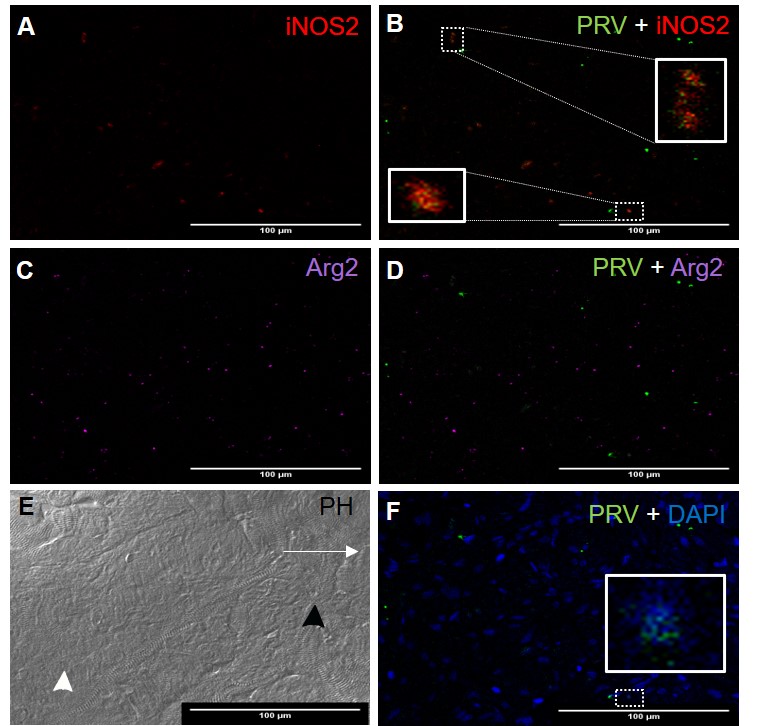

Supplement: Supplementary Figure 2 — Fluorescent in situ hybridization (FISH) of iNOS2, Arg2 and PRV-1 in HSMI (heart) at 4 wpc. (A) Few iNOS2 (red) positive M1 macrophages were detected. (B) Merged image showing co-localization of some PRV-1 in M1 macrophages (insets). (C) Widely distributed Arg2 (purple) positive M2 macrophages. (D) Merged image showing no co-localization of PRV-1 in M2 macrophages. (E) Phase contrast image showing all different layers of the heart i.e. epicardium (white arrow), compactum (black arrowhead) and spongiosum (white arrowhead). (F) PRV-1 (green) infected cells scattered in all different layers of the heart. Nuclei stained with DAPI (blue). Scale Bar = 100 µm. [file Image_2.jpeg]

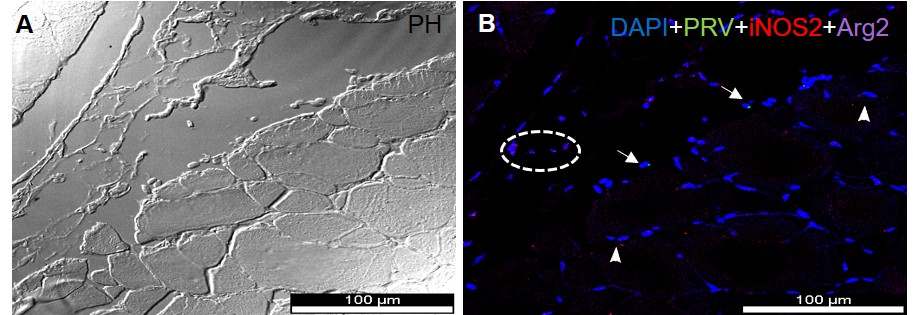

Supplement: Supplementary Figure 3 — Fluorescent in situ hybridization (FISH) of iNOS2, Arg2 and PRV-1 in HSMI (skeletal muscle) at 4 wpc. (A) Phase contrast image showing structure of myocytes. (B) Merged image showing PRV-1 infected cells (arrows) surrounded by M1 (arrowheads) and M2 macrophages (dotted circle). No co-localization of PRV-1 was observed in the M1 and M2 stained cells. Scale bar = 100µm. [file Image_3.jpeg]

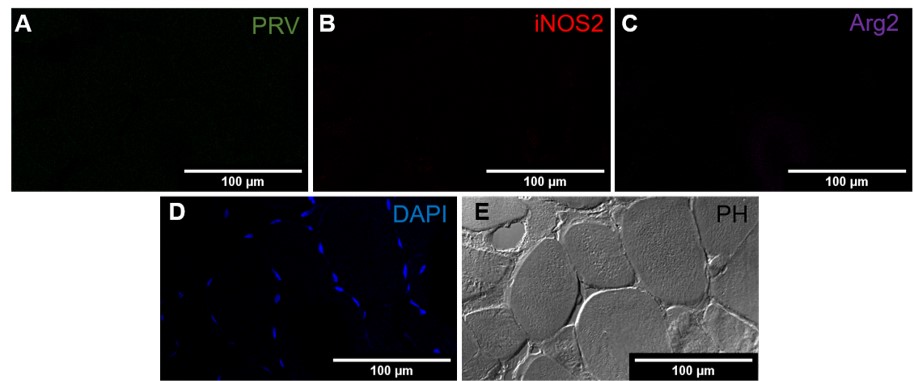

Supplement: Supplementary Figure 4 — Skeletal muscle tissue from un-infected control fish collected at a time corresponding to 4 wpc for the PRV-1 challenged fish. (A) No PRV-1 (green) detection, (B) No iNOS2 (red) positive cells (M1) were detected, (C) No Arg2 (purple) positive cells (M2) were detected. (D, E) Nuclei were stained with DAPI (blue) and phase contrast image shows myocytes. Scale bar = 100µm. [file Image_4.jpeg]

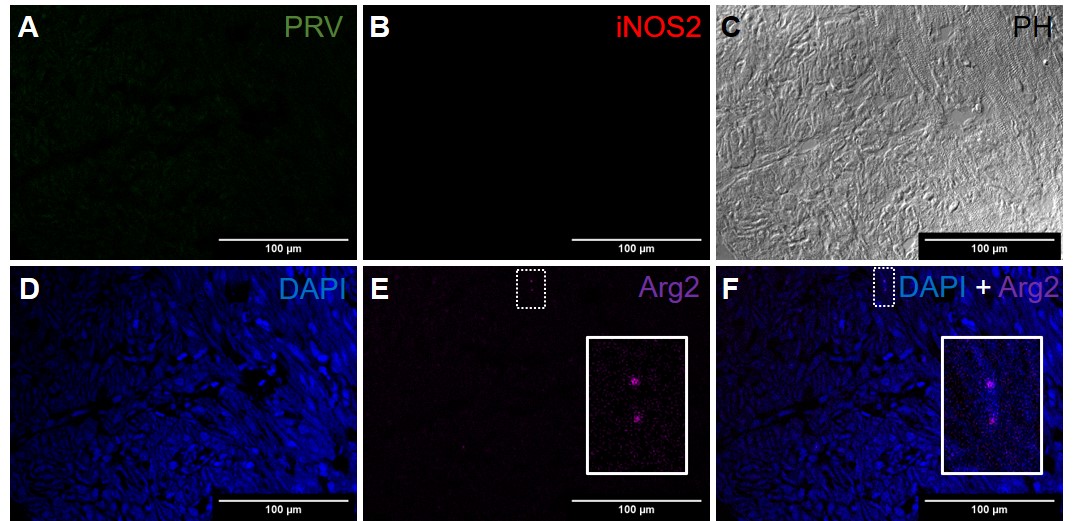

Supplement: Supplementary Figure 5 — Heart tissue from un-infected control fish collected at a time corresponding to 4 wpc for the PRV-1 challenged fish. (A) No PRV-1 (green) detection, (B) No iNOS2 (red) positive cells (M1). (C) Phase contrast image. (D) Cellular nuclei were stained with DAPI (blue). (E, F) Few Arg2 (purple) positive cells in the heart (insets). Scale bar = 100µm. [file Image_5.jpeg]

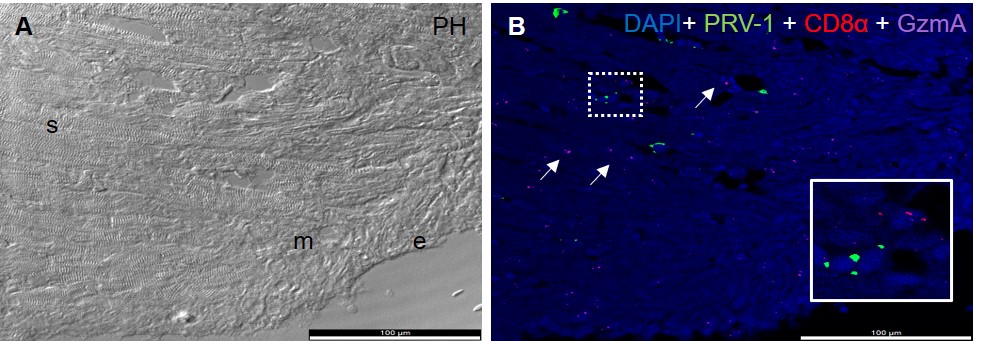

Supplement: Supplementary Figure 6 — Fluorescent in situ hybridization (FISH) of PRV-1, CD8α and GzmA in HSMI (Heart) at 4 wpc. (A) Phase contrast image showing structure of epi- (e), compactum (myo- (m)) and spongiosum layer. (B) Merged image showing no-colocalization of PRV-1 (green) but presence of few CD8+ (red) cells at the infected site (inset), whereas granzyme A (purple, arrows) specific transcripts are distributed elsewhere. Scale bar = 100 µm. [file Image_6.jpeg]

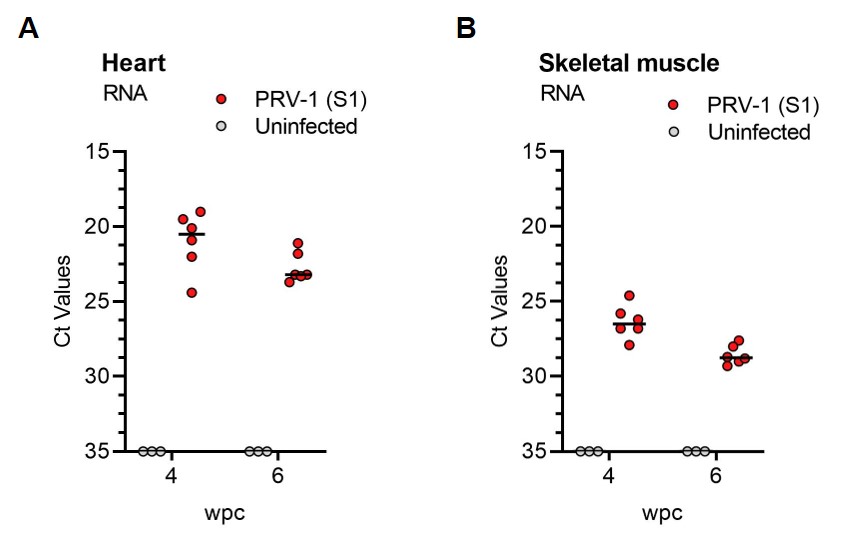

Supplement: Supplementary Figure 7 — RT-qPCR data showing median (line) PRV-1 level (red) in (A) heart and (B) skeletal muscle tissues at peak viral load (4 wpc) and peak pathological changes (6 wpc). Uninfected fish from each timepoint was included as a control fish. [file Image_7.jpeg]

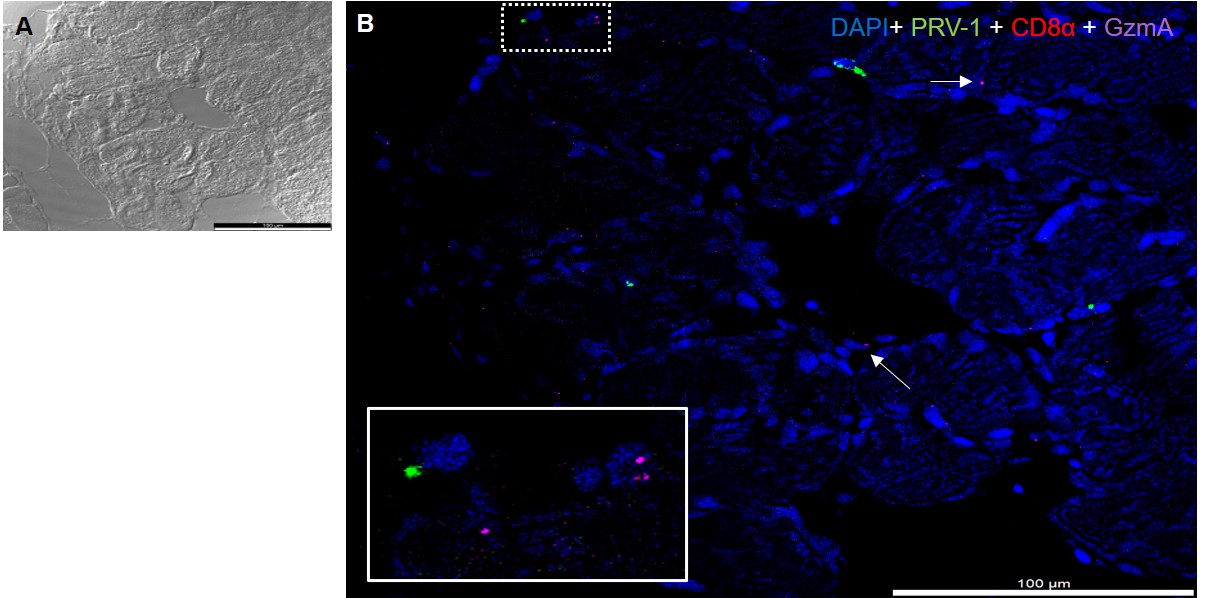

Supplement: Supplementary Figure 8 — Fluorescent in situ hybridization (FISH) of PRV-1, CD8α and GzmA in HSMI (skeletal muscle) at 4 wpc. (A) Phase contrast image showing myocytes structure. (B) Merged image showing PRV-1 (green) infected cells (inset) and sporadic presence of granzyme A (purple) specific cells (arrows). No CD8+ cells (red) were detected at 4 wpc. Scale bar = 100 µm. [file Image_8.jpeg]

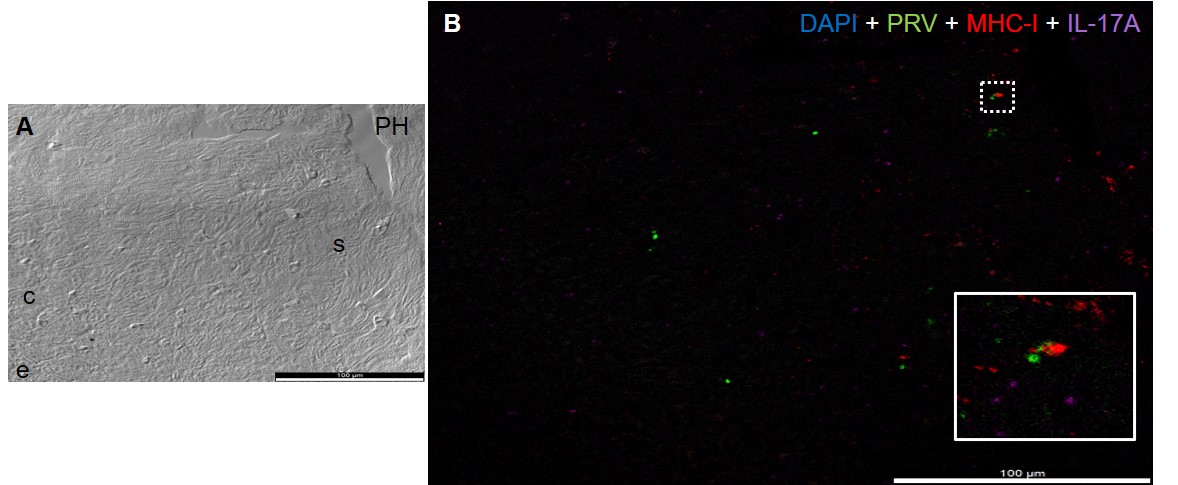

Supplement: Supplementary Figure 9 — Fluorescent in situ hybridization (FISH) of PRV-1, MHC-I and IL-17A in HSMI (Heart) at 4 wpc. (A) Phase contrast image showing structure of epi- (e), compactum (C) and spongiosum layer. (B) Merged image showing partial co-localization of PRV-1 (green) in MHC-I (red) positive cells. Very few IL-17A (purple) specific transcripts were detected (inset). Scale bar = 100 µm. [file Image_9.jpeg]

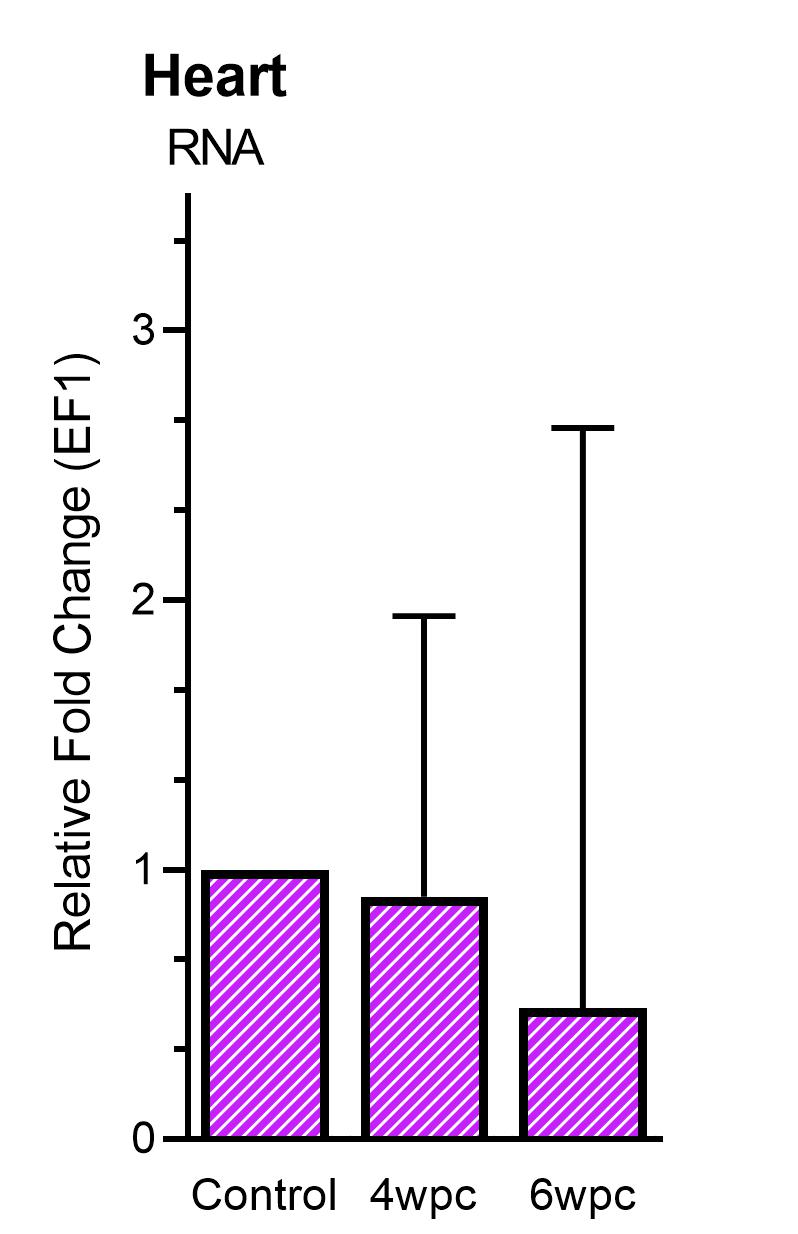

Supplement: Supplementary Figure 10 — Relative fold change expression (medians) of IL-17A at 4 wpc and 6 wpc in the heart tissue normalized against EF1α. [file Image_10.jpeg]

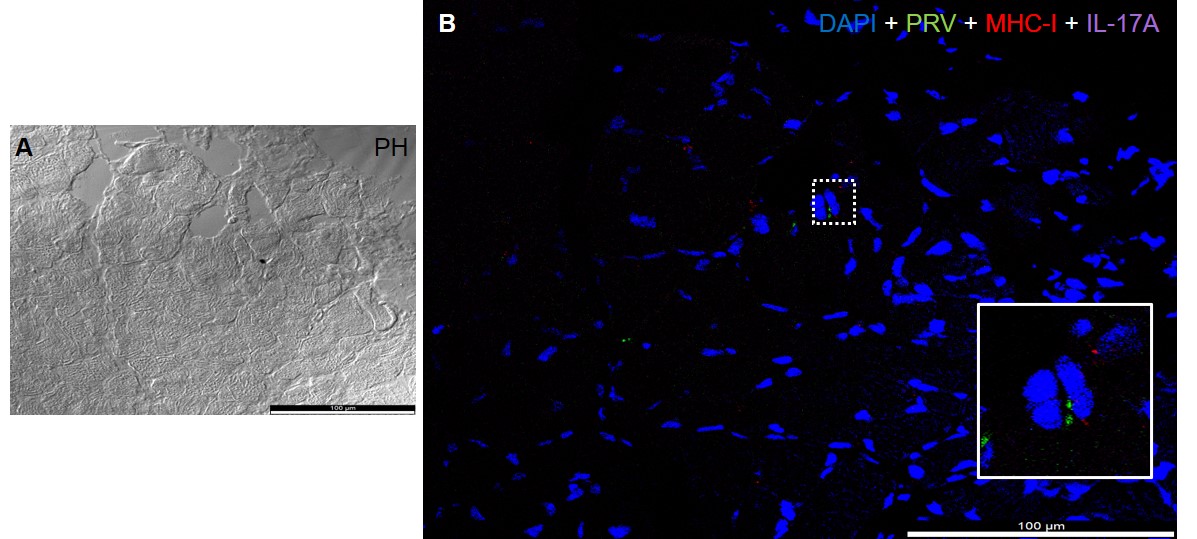

Supplement: Supplementary Figure 11 — Fluorescent in situ hybridization (FISH) of PRV-1, MHC-I and IL-17A in HSMI (skeletal muscle) at 4 wpc. (A) Phase contrast image showing myocytes structure. (B) Merged image showing PRV-1 (green) infected cells (inset) surrounded by MHC-I (red) cells. Scale bar = 100 µm. [file Image_11.jpeg]
